# Efficacy and Safety of OnabotulinumtoxinA for the Treatment of Platysma Prominence: A Systematic Review and Meta‐Analysis of Randomized Controlled Trials

**DOI:** 10.1111/jocd.70701

**Published:** 2026-01-29

**Authors:** Rahman Syed, Ameer Afzal Khan, Suleman Shah, Anfal Khan, Mohammad Idrees, Mohsin Ali, Mohammed Al Sinani, Mohammed Al Maqbali

**Affiliations:** ^1^ Department of Internal Medicine Swat Medical College Swat Pakistan; ^2^ Department of Internal Medicine Saidu Medical College Swat Pakistan; ^3^ Fatima College of Health Sciences Al Ain UAE; ^4^ Department of Metabolism, Digestion, and Reproduction Imperial College London London UK

**Keywords:** botulinum toxin type a, meta‐analysis, neck rejuvenation, OnabotulinumtoxinA, platysma prominence

## Abstract

**Background:**

Platysma prominence (PP) is a common aesthetic concern associated with aging, leading to visible neck bands and loss of jawline definition. OnabotulinumtoxinA has emerged as a minimally invasive treatment; however, data from randomized controlled trials (RCTs) remain fragmented.

**Aims:**

To systematically evaluate the efficacy and safety of onabotulinumtoxinA for treating moderate to severe PP through meta‐analysis of RCTs.

**Methods:**

PubMed, Cochrane CENTRAL, and ClinicalTrials.gov were searched from inception to March 2025 for RCTs comparing onabotulinumtoxinA with placebo in adults with PP. Two reviewers independently extracted data and assessed bias using the Cochrane RoB 2.0 tool. The primary outcomes were ≥ 1‐grade and ≥ 2‐grade improvement on the Clinician (C‐APPS) and Participant (P‐APPS) Allergan Platysma Prominence Scales. Secondary outcomes included patient satisfaction and treatment‐emergent adverse events (TEAEs). Random‐effects meta‐analysis was used to estimate pooled risk ratios (RRs) with 95% confidence intervals (CIs).

**Results:**

Three RCTs (*n* = 912) were included. OnabotulinumtoxinA significantly increased ≥ 1‐grade (RR = 4.11; 95% CI, 3.60–4.69) and ≥ 2‐grade (RR = 1.83; 95% CI, 1.54–2.17) improvements compared to placebo. Patient satisfaction was higher in the treatment group (RR = 5.55; 95% CI, 4.15–7.43). The incidence of TEAEs was similar between groups (RR = 0.95; 95% CI, 0.76–1.20), with most being mild and transient.

**Conclusions:**

OnabotulinumtoxinA is an effective and well‐tolerated minimally invasive option for improving platysma prominence, offering significant aesthetic and patient‐reported benefits without increasing adverse effects.

## 1 Introduction

Platysma prominence is a hallmark of cervical aging and jawline blunting, primarily driven by hyperfunctional platysma bands and progressive loss of lower facial support chemo‐denervation of the platysma using onabotulinumtoxinA has gained widespread use as a minimally invasive aesthetic intervention, using randomized controlled trials demonstrating meaningful improvements in neck contour and lower facial appearance with an acceptable safety profile [1, 2]. With age, repeated contraction of this muscle, loss of skin elasticity, descent of neighboring soft tissue, and changes in connective tissue architecture cause increased vertical neck bands (platysma prominence, PP) and diminished jawline definition [3, 4].

Platysma prominence is a common aesthetic concern. Individuals with moderate‐to‐severe PP frequently express disappointment, self‐consciousness, and a negative psychosocial impact due to the emergence of neck bands and jawline blunting [4, 5]. Traditional corrective methods include surgical operations (e.g., platysmaplasty, neck lift), which can result in remarkable contour improvement but come with downtime, cost, potential complications, and patient resistance [5].

In recent years, OnabotulinumtoxinA has emerged as a minimally invasive alternative for treating PP. The mechanism of action includes binding to presynaptic nerve terminals, internalization, cleavage of the SNAP‐25 protein (a component of the SNARE complex), suppression of acetylcholine release, and a brief reduction in muscular contraction [6, 7]. Clinically, it has been utilized in a variety of applications (wrinkles, hyperhidrosis, migraines), with platysma prominence being the most recently investigated [4].

Two key randomized, double‐blind, placebo‐controlled trials have shown that a single dose of OnabotulinumtoxinA (26–36 U or dose adjusted based on baseline PP) significantly improves PP severity (as assessed by investigators and participants) compared to placebo, with improvements visible as early as Day 14 post‐treatment [4, 8]. The phase 2 dosage‐ranging trial found that both “low dose” and “high dose” onabotulinumtoxinA groups achieved ≥ 1‐grade improvement on validated PP severity scores in about 75%–88% of treated participants versus ~12%–18% in placebo at Day 14. Adverse effects were usually moderate and temporary [8] The most recent phase 3 research indicated that 76.7% of treated subjects had ≥ 1‐grade improvement and 41.0% achieved ≥ 2‐grade improvement, compared to very low rates in the placebo group (*p* < 0.0001) [4]. Although promising, more synthesis is needed for long‐term safety, dose–response relationships, duration of benefit, and patient‐reported outcomes (such as satisfaction and psychological burden).

As a result, this paper includes a systematic review and meta‐analysis of randomized controlled trials (RCTs) that evaluated OnabotulinumtoxinA for the treatment of platysma prominence. Our objectives are to assess its efficacy (1‐grade and 2‐grade improvements in PP reported by clinicians and patients), safety profile, dose–response relationships, and patient‐centered outcomes to inform clinical practice.

## 2 Materials and Methods

This systematic review and meta‐analysis followed the Preferred Reporting Items for Systematic Reviews and Meta‐Analyses (PRISMA) guidelines [9]. The protocol was prospectively registered with the International Prospective Register of Systematic Reviews (PROSPERO; CRD420251127765) and followed the ethical guidelines specified in the Declaration of Helsinki [10].

A comprehensive literature search was conducted in PubMed, Cochrane Library, and ClinicalTrials.gov from the database's establishment to March 2025. The search strategy used the following keywords and Medical Subject Headings (MeSH): “OnabotulinumtoxinA” OR “Botox” AND “platysma prominence” OR “platysma bands” OR “neck aging” was tried on Cochrane, and the search strategy for PubMed and ClinicalTrials.gov is given below. The reference lists of the included studies and relevant reviews were additionally manually searched to find additional eligible papers.

Clinical trials.gov:

(“Platysma” OR “Neck Bands” OR “Lower Face Rejuvenation” OR “Jawline Contouring”) | (OnabotulinumtoxinA OR “Botulinum Toxin Type A” OR Botox).

PubMed:

(“Botulinum Toxins, Type A”[Mesh]) OR (Botulinum Neurotoxin Type A) OR ( *Clostridium botulinum* A Toxin) OR ( *Clostridium Botulinum* Toxin Type A) OR (Botulinum Neurotoxin A) OR (Neurotoxin A, Botulinum) OR (Botulinum A Toxin) OR (Toxin, Botulinum A) OR (Botulinum Toxin Type A) OR (Botulinum Toxin A) OR (Toxin A, Botulinum) OR (Oculinum) OR (Botox) OR (OnabotulinumtoxinA) OR (Onabotulinumtoxin A) OR (Meditoxin) OR (Vistabel) OR (Neuronox) OR (Vistabex) AND (platysma prominence) OR (platysma hypertrophy) OR (platysma muscle bands) OR (platysmal bands) OR (vertical neck bands) OR (prominent platysma) OR (neck bands) OR (platysmal laxity) OR (neck muscle prominence) OR (anterior neck bands) OR (neck skin folds) OR (neck contour abnormalities) OR (age‐related neck changes).

Studies were included if they were RCTs evaluating the efficacy of OnabotulinumtoxinA for platysma prominence, reported ≥ 1‐grade or ≥ 2‐grade improvement on validated scales (Clinician Allergan Platysma Prominence Scale [C‐APPS] or Participant Allergan Platysma Prominence Scale [P‐APPS]) and/or adverse events, and compared OnabotulinumtoxinA with placebo or active controls. Non‐RCTs (e.g., observational studies, case reports, or reviews) and studies that combined OnabotulinumtoxinA with other therapies (e.g., fillers) and did not have stratified outcomes were excluded.

Two reviewers (A.A.K., R.S.) independently screened titles, abstracts, and complete texts for eligibility, with conflicts addressed by consensus or a third reviewer (A.K.). Three reviewers (M.I., M.K., S.S.) extracted data independently using a standardized Excel template that included study characteristics (author, year, country, sample size), intervention details (dose in units, injection technique, follow‐up duration), and outcomes. Two reviewers independently assessed the risk of bias using the Cochrane Risk of Bias 2.0 (RoB‐2) technique, which included randomization, allocation concealment, blinding, completeness of outcome data, and selective reporting.

The primary outcomes were ≥ 1‐grade and ≥ 2‐grade improvement from baseline at maximal contraction, measured by (1) investigators using C‐APPS and (2) participants using P‐APPS. Both C‐APPS and P‐APPS use validated 5‐point photonumeric scales (1 = minimal, 2 = mild, 3 = moderate, 4 = severe, and 5 = extreme) to assess platysma prominence severity [5, 11]. Secondary outcomes included dose–response relationships, satisfaction levels (ANFLQ), and safety results (TEAEs and SAEs).

Meta‐analyses were carried out with RevMan 5.4 [12], which calculated risk ratios (RRs) with 95% confidence intervals (CIs) for dichotomous outcomes using fixed‐ or random‐effects models. The *I* ^2^ statistic was used to assess statistical heterogeneity, with criteria of < 25% for low heterogeneity, 25%–50% for moderate heterogeneity, 50%–75% for substantial heterogeneity, and > 75% for considerable heterogeneity. Because of the small number of included studies, no formal assessment of publication bias was performed using funnel plots or Egger's regression.

## 3 Results

**FIGURE 1:**
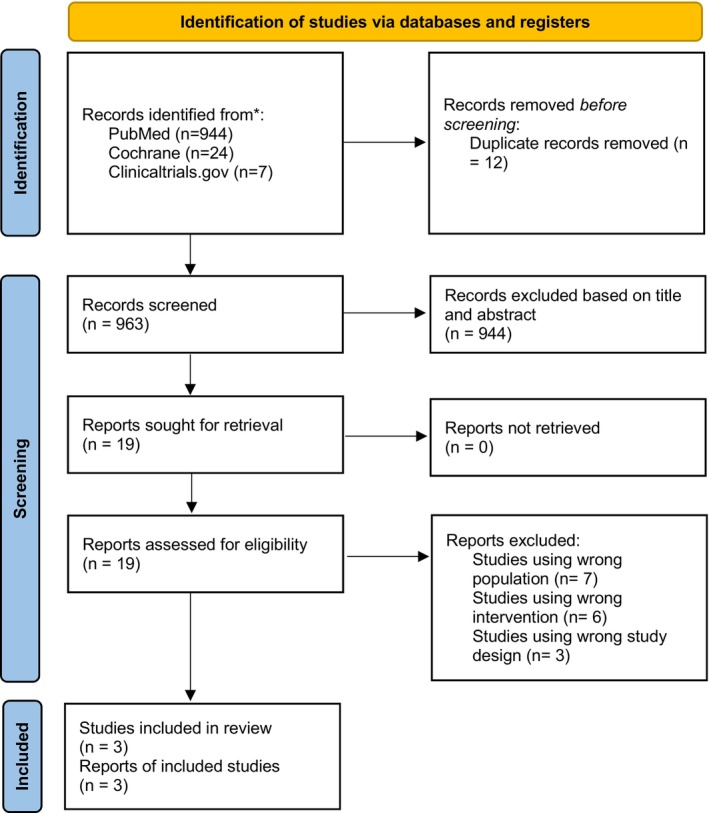
PRISMA flow diagram for study selection.

The initial search yielded 944 articles, of which three RCTs met the inclusion criteria, comprising a total of 912 participants (OnabotulinumtoxinA: *n* = 483; placebo: *n* = 429). Figure 1 shows the PRISMA flow diagram for study selection.

All trials compared OnabotulinumtoxinA with placebo using average doses of 26, 31, and 36 Units. One multicenter study by Rohrich et al. evaluated both low‐dose (26–36 U) and high‐dose (52–72 U) regimens [5]. All included trials were conducted across North America and Europe and were multicenter in design (Table 1).

**TABLE 1:** Characteristics of included studies.

Author	Country	Intervention	Control	Dose	*N* intervention	*N* control
Fabi (2025) [4]	US and Canada	onabotA	Placebo	26,31,36 U	186	181
Rohrich (2024) LD [5]	US and Canada	onabotA Low dose	Placebo	26,31,36 U	53	53
Rohrich (2024) HD [5]	US and Canada	onabotA High dose	Placebo	52, 62, 72 U	58	53
Shridharani et al. 2024 [8]	UK, US, Canada, Belgium, Germany	onabotA	Placebo	26, 31, or 36 U	186	195

**FIGURE 2:**
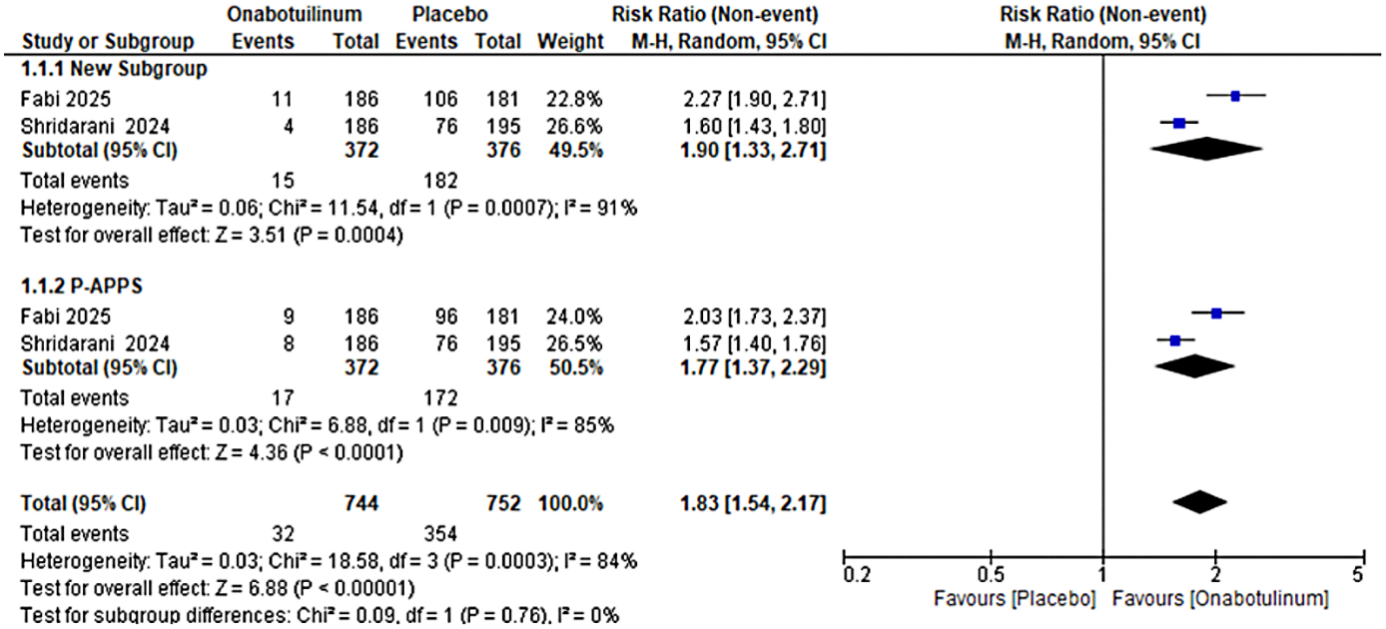
Forest plot showing the pooled effect of OnabotulinumtoxinA versus placebo for ≥ 2‐grade improvement in clinician‐rated platysma prominence severity (C‐APPS).

For ≥ 2‐grade improvement, pooled analysis of two studies demonstrated that OnabotulinumtoxinA significantly improved platysma prominence compared with placebo, with an RR of 1.90 (95% CI: 1.33–2.71) for investigator‐assessed outcomes (C‐APPS) and 1.77 (95% CI: 1.37–2.29) for participant‐assessed outcomes (P‐APPS). The overall combined estimate yielded an RR of 1.83 (95% CI: 1.54–2.17), although considerable heterogeneity was observed, as shown in Figure 2. Grade analysis revealed moderate quality of evidence (as the studies assess directness, lack publication bias, low risk of bias, and high heterogeneity).

**FIGURE 3:**
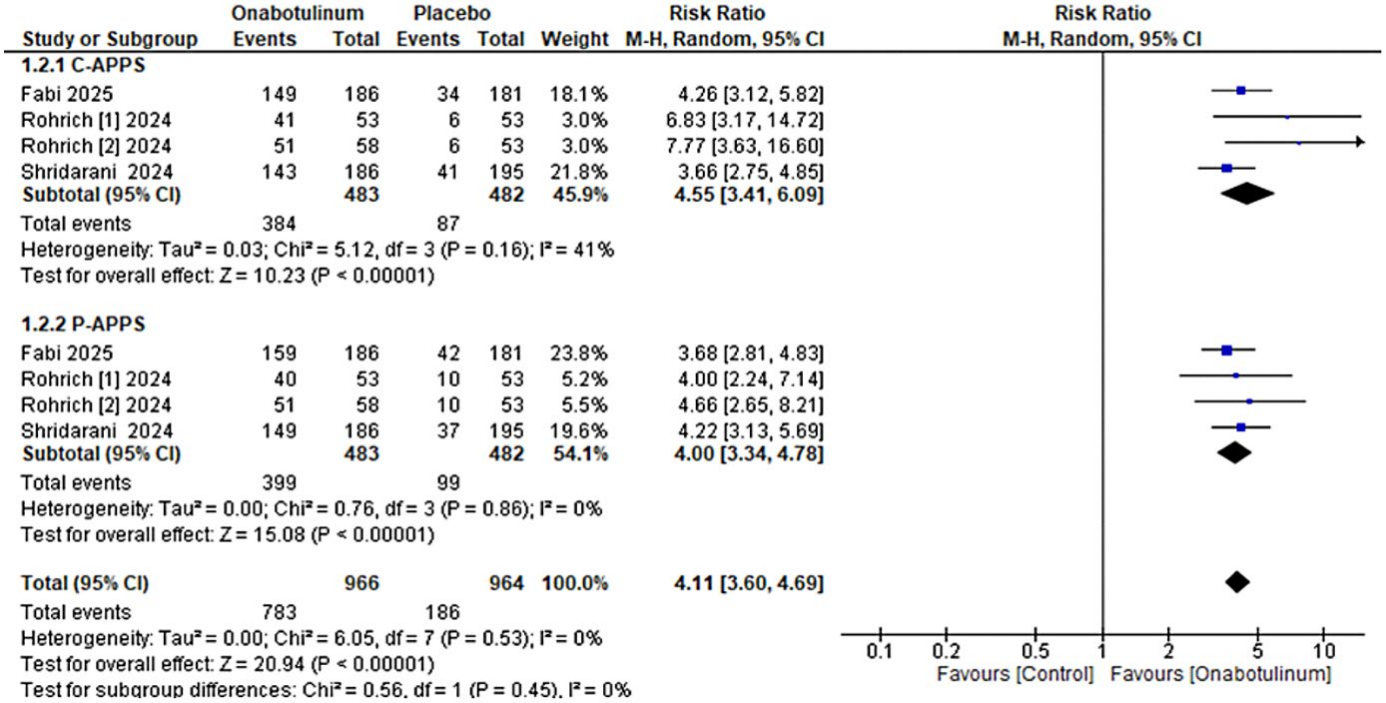
Forest plot of ≥ 1‐grade improvement in platysma prominence severity (C‐APPS and P‐APPS).

For ≥ 1‐grade improvement, pooled analysis across all studies showed an RR of 4.55 (95% CI: 3.41–6.09) for C‐APPS with moderate heterogeneity and 4.00 (95% CI: 3.78–4.78) for P‐APPS. The overall pooled RR was 4.11 (95% CI: 3.60–4.69) with negligible heterogeneity, demonstrating a robust treatment effect, as shown in Figure 3. Grade analysis revealed high certainty of evidence (low heterogeneity, assessed directness, low risk of bias and no publication bias).

**FIGURE 4:**
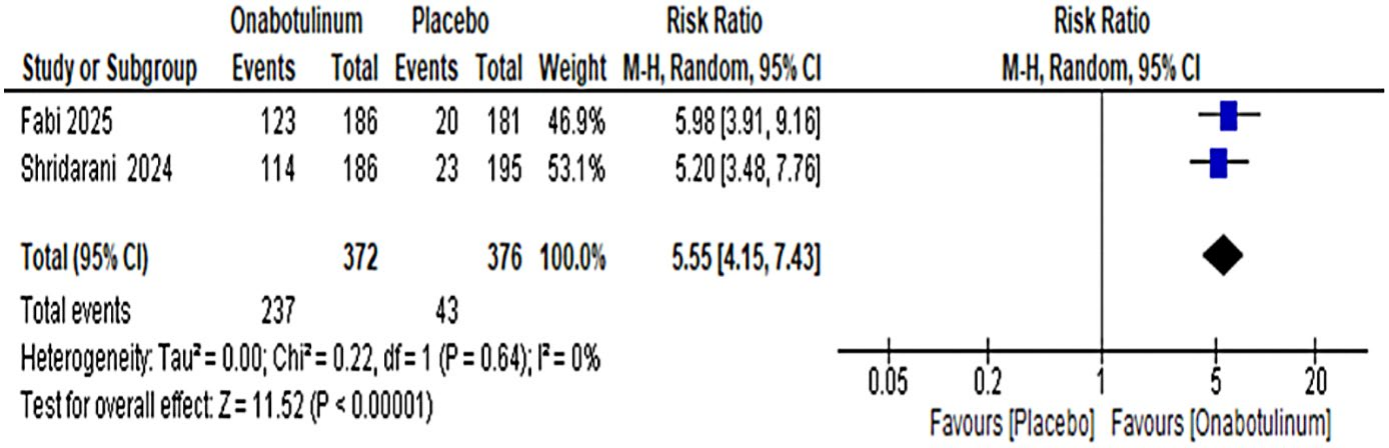
Patient satisfaction following OnabotulinumtoxinA versus placebo treatment.

Regarding secondary outcomes, two studies using the ANFLQ questionnaire reported significantly higher satisfaction rates (“satisfied” or “very satisfied”) in the OnabotulinumtoxinA group compared with placebo (RR: 5.55, 95% CI: 4.15–7.43) with no heterogeneity and high to moderate certainty of evidence. Safety analysis showed that treatment‐emergent adverse events occurred in 109 of 483 participants in the OnabotulinumtoxinA group versus 114 of 429 in the placebo group (RR: 0.95, 95% CI: 0.76–1.20), indicating no significant difference between groups. Treatment‐related adverse events were also comparable (33 vs. 31 events; RR: 1.04, 95% CI: 0.65–1.67) (Figure 4). Serious adverse events were rare, occurring in four placebo recipients and two OnabotulinumtoxinA recipients. The most common adverse events were mild injection‐site hemorrhage and bruising. Both the TEAE and SAE had a high certainty of evidence using grade analysis.

**FIGURE 5:**
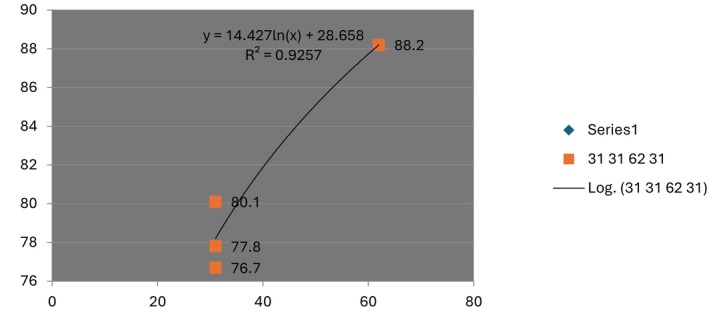
Dose–response relationship between OnabotulinumtoxinA dose and efficacy.

Due to limited data points, a formal meta‐regression was not performed to assess the dose–response relationship. However, scatterplot analysis suggested a positive correlation between dose and efficacy, with an *R* ^2^ value of 0.925, indicating that higher doses of OnabotulinumtoxinA may be associated with greater treatment effects; but due to limited data points, the association between the dose and response cannot be generalized, as shown in Figure 5.

**FIGURE 6:**
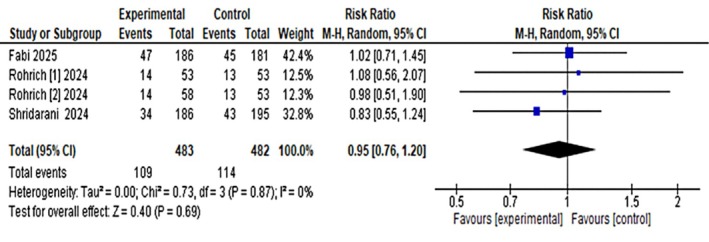
Pooled analysis of treatment‐emergent adverse events (TEAEs).

**FIGURE 7:**
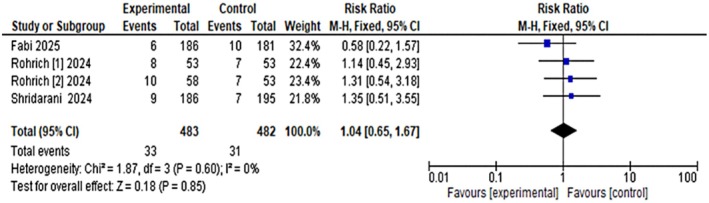
Pooled analysis of treatment‐related adverse events.

Pooled analysis of all included trials showed that treatment‐emergent adverse events (TEAEs) occurred in 109/483 (22.6%) participants receiving OnabotulinumtoxinA versus 114/429 (26.6%) receiving placebo, with no significant difference between groups (RR = 0.95; 95% CI: 0.76–1.20). When limited to treatment‐related TEAEs, 33 events were reported in the OnabotulinumtoxinA group and 31 in the placebo group (RR = 1.04; 95% CI: 0.65–1.67) (Figures 6 and 7). Most events were mild, transient injection‐site reactions; serious adverse events were rare and comparable between groups.

**FIGURE 8:**
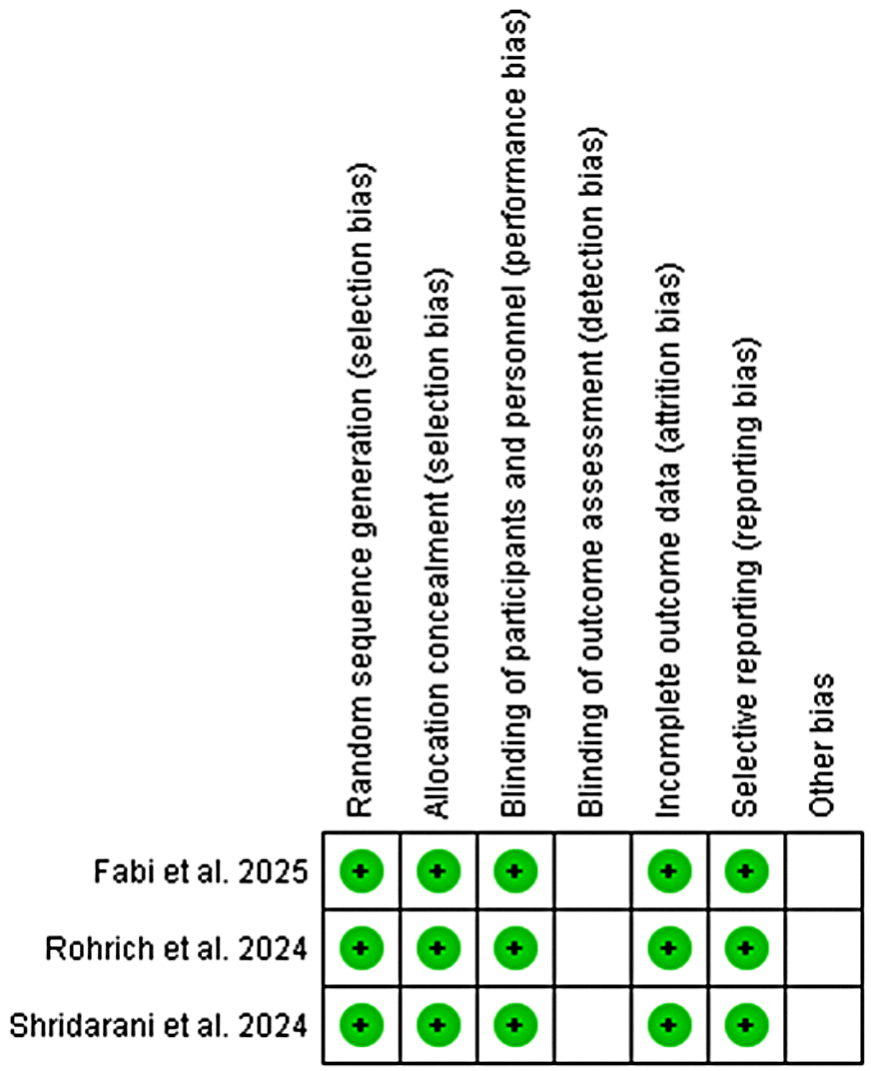
Risk of bias summary (RoB 2.0).

All the studies were low risk double‐blinded studies except for the blinding of outcome assessment as this domain had some concerns. The drug has clearer and measurable biological outcomes (muscle paralysis); participants will report a reduction in muscle contraction compared to placebo. The risk of bias is summarized in the Figure 8.

## 4 Discussion

This systematic review and meta‐analysis of randomized controlled trials presents the most compelling evidence yet that onabotulinumtoxinA is an effective and well‐tolerated nonsurgical treatment for moderate to severe platysma prominence (PP). OnabotulinumtoxinA treatment resulted in significantly higher rates of ≥ 1‐grade and ≥ 2‐grade improvement on the Clinician Allergan Platysma Prominence Scale (C‐APPS) and Participant Allergan Platysma Prominence Scale (P‐APPS) compared to placebo.

### 4.1 Efficacy and Dose Response

Our analysis shows a clear dose–response pattern, with higher dosages resulting in a higher proportion of responders for ≥ 2‐grade improvement while remaining safe. The phase 2 dosage‐ranging study by Rohrich et al. found ≥ 1‐grade improvement in 77.8% of participants at low dose and 88.2% at high dose, compared to 12.0% with placebo at Day 14 [5]. In a phase 3 trial, Fabi et al. found that 76.7% of patients achieved ≥ 1‐grade improvement and 41% achieved ≥ 2‐grade improvement on Day 14, compared to very low rates in the placebo [4]. Shridharani et al. corroborated similar findings in a multicenter phase 3 RCT, observing both improved investigator and patient outcomes [8]. Taken together, our findings support individualized dosing based on baseline severity and patient expectations, as recommended by expert consensus guidelines [13, 14].

### 4.2 Safety and Adverse Events

Our meta‐analysis revealed reassuring safety findings. The pooled incidence of treatment‐emergent adverse events (TEAEs) was comparable across the onabotulinumtoxinA and placebo groups (RR 0.95; 95% CI 0.76–1.20), and treatment‐related TEAEs were similarly not substantially different. The majority of adverse effects were moderate and temporary, including injection‐site hemorrhage and bruising, which is consistent with the known safety profile of botulinum toxin injections [4, 5, 15]. Serious adverse effects were uncommon and comparable across groups, with no evidence of systemic toxin dissemination. These findings are consistent with previous systematic reviews of botulinum toxin for platysmal bands and neck rejuvenation, which have found minimal complication rates and primarily modest, local effects [15, 16].

### 4.3 Anatomical and Technique Considerations

Injection method and anatomical precision are important predictors of efficacy and safety. Studies emphasize the necessity of adhering to platysma motor end‐plate distribution, injection depth, and dose per point in order to optimize outcomes and minimize side effects such as neck weakness or dysphagia [15, 16]. The “Nefertiti lift” method and other lower‐face rejuvenation treatments have demonstrated that targeted botulinum toxin injections can result in significant aesthetic improvement with minimal downtime [13, 17]. Incorporating such anatomical criteria into everyday therapy may improve the therapeutic index of onabotulinumtoxinA in PP patients.

### 4.4 Limitations and Future Directions

Several limitations require consideration. First, the majority of the trials included only examined a single injection session with a follow‐up of 120 days, which limits inferences concerning long‐term durability, retreatment intervals, and cumulative safety. Second, the study's demographic was largely female and White, restricting its applicability to men and people with more diverse complexion types. Third, variations in dosage regimens, injection techniques, and patient‐reported outcomes could have altered aggregate effect estimates. Fourth, the publication bias of the studies was not assessed which was due to the limited number of studies that would limit the power of the findings. And finally, patient satisfaction ratings were frequently secondary objectives with inconsistent standardization across research.

Future research should fill these gaps by performing randomized trials with several treatment cycles and longer follow‐up periods, involving more diverse populations, and standardizing patient‐reported outcome measures. Comparative effectiveness studies of onabotulinumtoxinA vs. surgical interventions or other non‐invasive methods, as well as dose‐optimization studies, are required. Moreover, the study demographics need to be explored for men and blacks, as the current studies only focus on females and whites. High‐resolution anatomical mapping of the platysma could help to modify injection techniques to maximize efficacy while minimizing side effects.

## 5 Conclusion

This meta‐analysis confirms onabotulinumtoxinA as an effective, well‐tolerated, and minimally invasive treatment for platysma prominence. Both clinicians and patients reported significant improvements in severity and satisfaction, with no rise in serious adverse events. Tailoring the dose to the baseline severity and following anatomical injection recommendations may improve outcomes even further. To properly define the role of onabotulinumtoxinA in lower‐face and neck aesthetics, further study should be conducted into long‐term efficacy, optimal retreatment regimens, and larger patient groups.

## Author Contributions

Rahman Syed and Ameer Afzal Khan conceived and designed the study, performed data extraction, and drafted the initial manuscript. Suleman Shah and Mohammed Al Maqbali contributed to data interpretation, literature review, and manuscript editing. Anfal Khan, Mohammad Idrees, and Mohsin Ali assisted in data collection, statistical analysis, and figure preparation. Mohammed Al Sinani critically reviewed the manuscript for intellectual content and provided expert input on methodology and clinical relevance. All authors read and approved the final version of the manuscript and agree to be accountable for all aspects of the work.

## Ethics Statement

This study was exempt from IRB as this was a systematic review.

## Conflicts of Interest

The authors declare no conflicts of interest.

## Data Availability

The data that support the findings of this study are available from the corresponding author upon reasonable request.
